# Impact of treatment duration on the effectiveness of opioid analgesia: a systematic review and meta-analysis

**DOI:** 10.3399/BJGP.2025.0705

**Published:** 2026-07-14

**Authors:** Liesbeth S Froentjes, Catherine AM Barrington, Rodger A Craig, Mareiz Morcos, Dawn M Wedman, Christina S Korowynk, Michael D Kelmer, Farah N Campbell, Scott R Garrison

**Affiliations:** 1 School of Public Health, University of Alberta, Edmonton, AB, Canada; 2 Department of Emergency Medicine, Covenant Healthcare College of Medicine, Central Michigan University, Mount Pleasant, MI, US; 3 Department of Anesthesiology and Pain Medicine, University of Alberta, Edmonton, AB, Canada; 4 Faculty of Pharmacy, University of Waterloo, Waterloo, ON, Canada; 5 Department of Family Practice, University of British Columbia, Vancouver, BC, Canada; 6 Department of Family Medicine, University of Alberta, Edmonton, AB, Canada

**Keywords:** chronic pain, duration of treatment, low back pain, opioids, osteoarthritis, primary health care.

## Abstract

**Background:**

Opioid use can heighten pain perception over time.

**Aim:**

To determine whether the effectiveness of opioid analgesia diminishes with treatment duration in those with chronic low back pain or osteoarthritis.

**Design and setting:**

Systematic review of randomised trials comparing opioids with placebo/opioid-minimised pain management strategies in those with chronic low back pain or osteoarthritis.

**Method:**

A random-effects meta-analysis using MEDLINE, Embase, CENTRAL, and Scopus databases was undertaken in January 2025. The primary outcome was attainment of clinically important pain relief (‘moderate’ or ≥30% improvement). The secondary outcome was on-treatment pain (100-point scale). The primary analysis was difference in primary/secondary outcomes across short-term (≤4 weeks), intermediate-term (4–12 weeks), and long-term (≥12 weeks) duration subgroups.

**Results:**

In total, 27 trials were eligible for inclusion. Clinically important pain relief differed significantly between short-, intermediate-, and long-term treatment durations (*P* = 0.05). Opioid recipients were more likely to be responders in short-term trials (eight trials, risk ratio [RR] 1.42, 95% confidence interval [CI] = 1.08 to 1.89, moderate-certainty evidence), but not in intermediate-term trials (three trials, RR 1.04, 95% CI = 0.84 to 1.30, low-certainty evidence), or long-term trials (nine trials, RR 0.91, 95% CI = 0.73 to 1.14, moderate-certainty evidence), which trended in favour of controls. The mean difference in pain scores failed to reach this review’s definition of clinical significance (≥10 points) for any timepoint, but was statistically significant in the short and intermediate term.

**Conclusion:**

Although opioids likely provide meaningful pain relief over short durations (≤4 weeks), they appear to provide little or no benefit beyond placebo over longer periods, and may worsen pain control beyond 12 weeks.

**How this fits in**

Although opioids are commonly prescribed for the management of pain, their use can lower the threshold for pain perception. To the authors’ knowledge, this is the first meta-analysis of opioids versus placebo/opioid-minimising pain management strategies that examines the effectiveness of opioid analgesia over time. Although opioids provided meaningful short-term pain relief (≤4 weeks), they provided little or no pain relief over longer periods, and may have worsened pain control beyond 12 weeks. Although opioids are effective for the management of acute pain, clinicians should reconsider offering them to patients whose pain is expected to be chronic.

## Introduction

Although opioids are commonly prescribed for the management of pain, there is evidence that opioid use increases pain sensitivity, an effect known as opioid-induced hyperalgesia, which could theoretically produce a counterproductive feedback loop of upwards dose titration and exacerbation of pain.^1^ The adequacy of pain relief could also be lessened by the development of opioid tolerance, opioid dependence, or withdrawal symptoms.^2,3^ If such counterproductive feedback loops do indeed exist, the adequacy of pain relief obtained from opioids may depend on the duration of treatment.

To determine whether the effectiveness of opioid pain relief diminishes over time the authors systematically identified all randomised controlled trials (RCTs) comparing opioid with non-opioid pain management strategies for the treatment of osteoarthritis (OA) or chronic low back pain, and compared the ability of opioids to produce clinically meaningful pain relief over the short, intermediate, and longer term.

## Method

This review was registered with PROSPERO (ID: CRD42020147459). The full protocol is available in Supplementary Information S1, and the algorithm for the 22 January 2025 search of MEDLINE, Embase, CENTRAL, and Scopus (with no language restriction) is available in Supplementary Information S2.

### Eligibility criteria

Eligible studies were randomised trials with a parallel or crossover design that enrolled participants with OA or chronic low back pain:

- with an opioid intervention arm;
- used either placebo or opioid-minimised pain management as a control; and
- included either a responder analysis, a 100 mm visual analogue pain scale (VAS), or an 11-point (0–10) numerical rating scale (NRS) for pain among their efficacy outcomes.

A responder analysis reports the proportion of participants achieving a predetermined threshold of improvement. Opioid-minimised pain management, as defined by the authors in the current study, begins with non-opioid methods of pain relief and uses opioids, if at all, after all non-opioid treatment options have been exhausted.

Trials were excluded that:

- exclusively enrolled participants with back pain resulting from surgery, cancer, neurogenic claudication, radiculopathy, sciatica, or rheumatic conditions (for example, rheumatoid arthritis or ankylosing spondylitis);
- required participants to have opioid-use disorder;
- utilised only non-self-administered opioid formulations (for example, IV infusion);
- provided opioid rescue medication to control participants; or
- provided non-opioid analgesics in the opioid arm (for example, acetaminophen) without the same being available to all treatment groups.

For the analysis of pain scores, additionally trials were excluded that only provided a per-protocol analysis. The study also excluded trials if they only examined opioids claiming non-opioid mechanisms of pain relief (these being tramadol and tapentadol). This exclusion was made because the goal was to determine whether opioid efficacy wanes over time and the inclusion of agents with dual modes of action would have confounded this assessment. Separate from their effect on opioid receptors, tramadol is a serotonin–norepinephrine reuptake inhibitor, and tapentadol is a norepinephrine reuptake inhibitor.^4,5^

In addition, the study excluded all trials that employed an ‘enriched enrolment randomised withdrawal’ (EERW) design before any data were analysed. This design was previously unknown to the authors and hence not addressed in the original review protocol. EERW designs provide run-in opioid medication to all participants for a number of weeks, randomise only those who respond to opioids, and then withdraw opioids from the control group. This trial design was excluded because of a very high risk of bias — specifically, high risk of selection bias, unblinding of control participants as they withdraw from opioids, and the potential for development of physical dependence or opioid-induced hyperalgesia during the run-in period, all of which may exacerbate pain in control participants. Post hoc, the authors separately examined whether EERW trials produce meaningfully different results from traditional RCTs.

### Study selection and data collection

Two authors independently performed title and abstract screening, full-text screening, and data extraction. Any discrepancies were adjudicated by a third author. In the case of missing or unclear data an attempt was made to contact authors or trial sponsors for additional information.

### Risk of bias assessments

Three authors independently assessed potential bias using the 2011 Cochrane Collaboration risk of bias tool, which has authors rate the risk of bias as ‘low’, ‘unclear’, or ‘high’ in the areas of: random sequence generation; allocation concealment; blinding of participants and personnel; blinding of outcome assessment; incomplete outcome data; selective reporting; and other bias.^6^ Quality of evidence was assessed using the Grading of Recommendations Assessment, Development, and Evaluation approach,^7^ and risk of publication bias across trials was assessed through visual inspection of funnel plots for the primary and secondary analyses.

### Outcomes

The primary outcome was the number of participants that achieved a minimal clinically important difference (MCID) in pain, which consensus guidelines define as ‘moderate’ or ≥30% improvement in pain from baseline.^8^ When this measure was not reported, or when multiple responder outcomes were available, a predefined outcome hierarchy was employed (see Supplementary Box S1) to select the responder outcome closest to a ≥30% improvement. The hierarchy makes use of the authors’ experience that typical patients enrolled in pain studies have baseline pain scores of approximately 5–6 out of 10. Hence a change of ≥2, or an absolute score of ≤4, would represent roughly 30% improvement.

The secondary outcome was self-reported pain scores as rated on either a 100 mm VAS or 11-point NRS. The authors of the current study preferentially used on-treatment pain measures over change from baseline, and used a 100 mm VAS over an 11-point NRS if both were available. The authors also preferentially used pain outcomes assessing average daily pain but accepted other ways of defining pain such as average pain over the preceding week(s) or averaged worst daily pain. All outcomes were converted to a 100-point scale and a between-group difference of 10 points was considered to be the MCID as per consensus guidelines.^8^ Although in the study protocol the authors also prespecified the intention to examine the per cent increase from baseline in opioid dose across the three duration subgroups, this information was not available in the eligible trials.

### Data analysis

All meta-analyses were performed using Review Manager (version 5.3), used a random-effects model, and assessed heterogeneity using the *I* ^2^ statistic.

#### Primary analysis: effect of trial duration

To explore variation in treatment effect over time, eligible trials were divided into three duration subgroups: short term (≤4 weeks), intermediate term (4–12 weeks), and long term (≥12 weeks), as per the common literature definition of acute (<4 weeks) and chronic pain (>3 months).^1,9,10^

When trials compared multiple eligible opioid formulations, each with its own parallel arm, results were considered from each opioid arm separately, and corresponding control groups were created by dividing both the total number of control participants and the number of responders in the control group evenly between arms. For trials with multiple arms of the same opioid at different doses/dosing schedules, the treatment arms were combined. When available, the modified intent-to-treat (mITT) population was used, defined as all randomised patients who received ≥1 dose of study medication. When this population was not reported all randomised patients were included. In the current study, the authors considered all missing patients in the author-reported responder analysis, aside from those excluded from the mITT population, as ‘non-responders’.

#### Sensitivity analysis: effect of the EERW trial design

For the primary (responder) outcome the authors also examined whether the trials that were excluded because of an EERW trial design produced meaningfully different results from the included trials. This was done by including all the previously excluded EERW trials in a new forest plot with both EERW and ‘traditional’ RCT subgroups.

## Results

### Study selection

**Figure 1.**
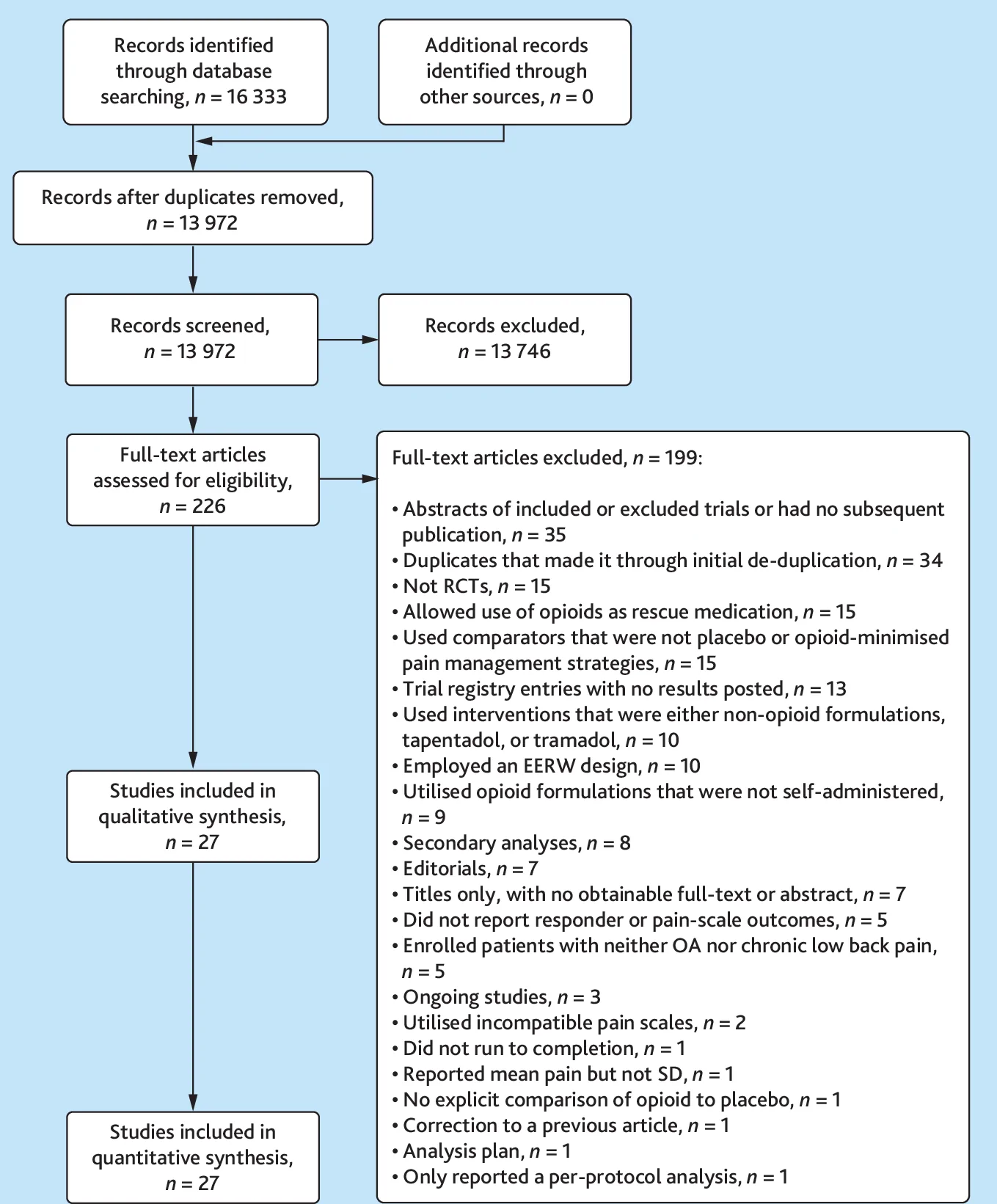
Study flow diagram. EERW = enriched enrolment randomised withdrawal. OA = osteoarthritis. RCT = randomised controlled trial. SD = standard deviation.

The de-duplicated title and abstract search yielded 13 972 articles, of which 226 underwent full-text review, and 199 were excluded, leaving 27 eligible trials.^11–37^ Detailed reasons for exclusion are provided in Figure 1. Of note, 10 trials were excluded because of an EERW design.^38–47^ Individual reasons for exclusion, and a list of included trials can be found in Supplementary Information S3.

### Study characteristics

Key trial characteristics are provided in Supplementary Table S1, with more detailed descriptions in Supplementary Box S2. Of 27 included trials, 12 were long term,^11,13,14,17,23,25,27,29,30,33,35,36^ two were intermediate term,^24,34^ and 13 were short term.^12,15,16,18–22,26,28,31,32,37^ All but two short-term crossover trials employed parallel designs.^19,32^

Nineteen trials enrolled participants with OA,^11,13,15,16,20–22,24–31,33–35,37^ seven enrolled participants with chronic low back pain,^12,14,17–19,32,36^ and one enrolled a mixed population.^23^ Across all included trials, participants were 57.5% female, and had a mean age of 57.3 years. Most trials (*n* = 24/27) included both opioid-naïve and opioid-experienced participants (often with limitations on maximum dosage or frequency of use), while two did not specify,^12,22^ and one required opioid experience.^19^ Oxycodone was used in 13 of 27 trials (48%).^11,14,16,20,25–27,31–34,36,37^ Other opioids included buprenorphine,^13,19,28^ oxymorphone,^21,26^ hydromorphone,^30,35^ transdermal fentanyl,^24,29^ morphine,^15,18^ and the remaining used either ADL5747 or ADL5859 (δ-opioid receptor agonists),^31^ cebranopadol,^17^ codeine/acetaminophen,^22^ propoxyphene,^12^ or ‘general opioid therapy’,^23^ which meant the opioid was selected according to prescriber/participant preferences. Most trials (*n* = 24, 89%) compared with placebo. The remaining three compared with a) the prescriber’s choice of a variety of non-opioid medications (simultaneously available to those in the opioid arm);^23^ b) tolterodine (a potentially side effect-producing ‘active placebo’);^32^ and c) acetaminophen.^22^ Characteristics of the excluded EERW trials included in the sensitivity analysis are in Supplementary Box S3.

### Risk of bias

**Figure 2.**
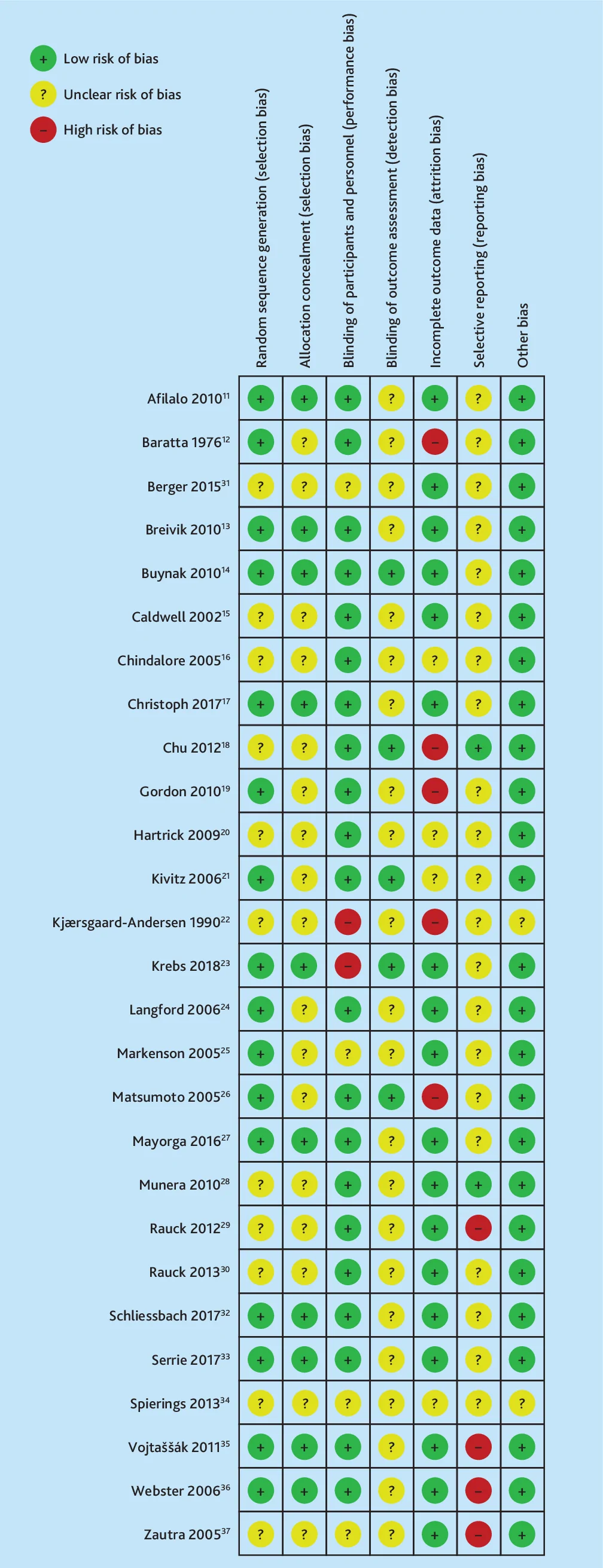
Risk of bias for all included studies.

Risk of bias for all included trials is summarised in Figure 2 and risk of bias for individual analyses is provided in Supplementary Figure S1. Detailed risk of bias assessments can be found in Supplementary Box S4. A common issue across included trials was unclear reporting, specifically regarding the blinding of outcome assessors.

**Figure 3.**
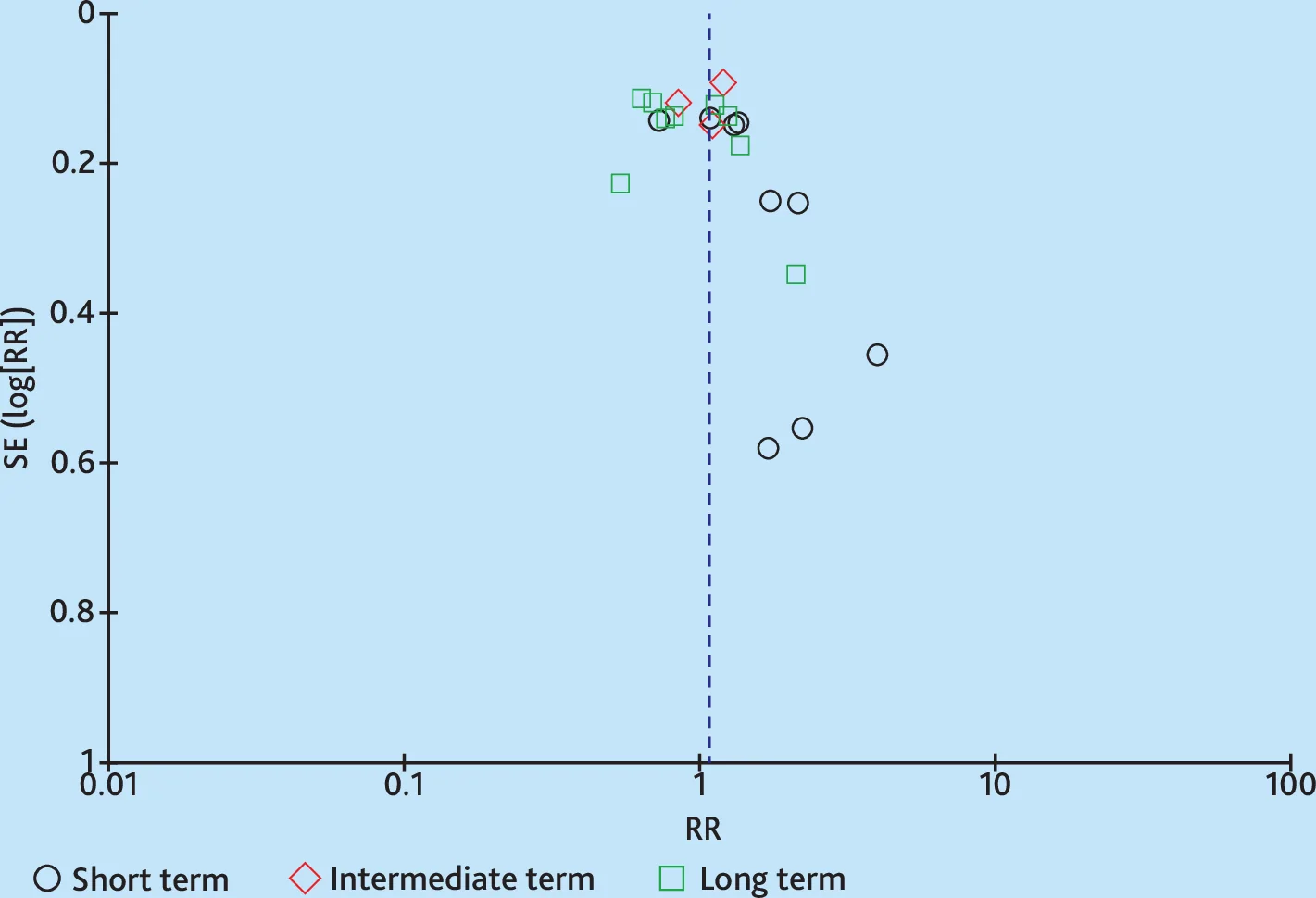
Funnel plot for studies contributing to the primary (responder) analysis for attainment of a ≥30% reduction in pain from baseline, opioid versus control, over short-, intermediate-, and long-term treatment durations. RR = risk ratio. SE = standard error.

Visual inspection of the funnel plot for the responder analysis (Figure 3) suggests smaller trials that are less favourable to opioids are missing, indicating probable publication bias in favour of opioids. Similar opioid-favouring publication bias is also evident in the assessment of pain scores (see Supplementary Figure S2).

### Analgesic efficacy over time

#### Primary outcome: number of responders

**Figure 4.**
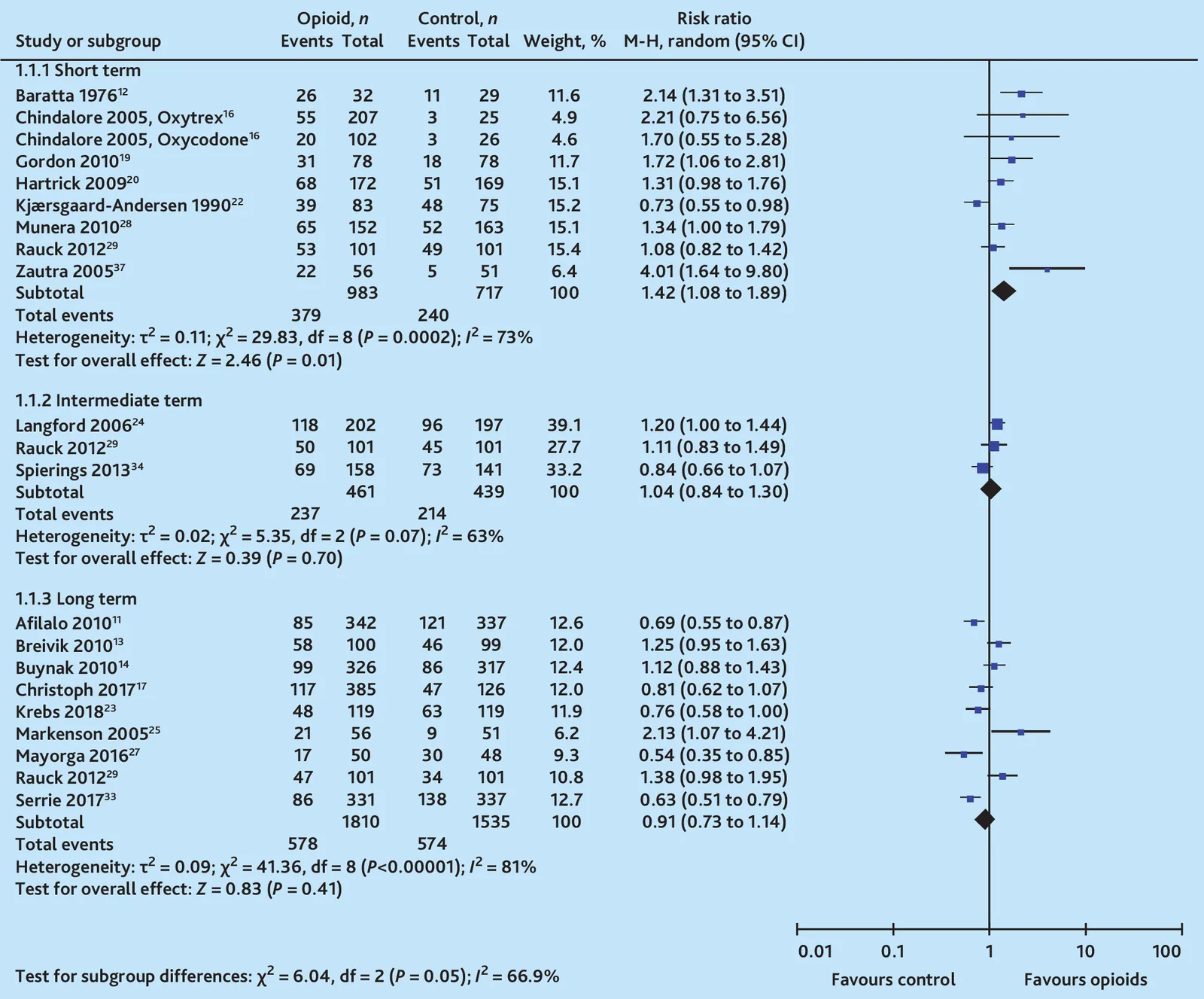
Forest plot for attainment of a meaningful (≥30%) reduction in pain, opioids versus control, over short-, intermediate-, and long-treatment durations. Ordered from shortest to longest interval of assessment. df = degrees of freedom. M-H = Mantel–Haenszel method.

The analgesic efficacy of opioids compared with control differed significantly between short-, intermediate-, and long-term trials (χ² = 6.04, degrees of freedom [df] = 2, *P* = 0.05). Opioids displayed superior pain relief only in short-term trials, with long-term trials trending towards fewer of the participants reporting meaningful pain relief in the opioid arm (Table 1 and Figure 4).

**Table 1.** Summary of findings for primary and secondary outcomes by treatment duration. Population: patients with OA and chronic low back pain; intervention: opioid; comparison: placebo or opioid-minimised pain management strategies

**Subgroup**	**Average treatment duration (duration analysis), weeks**	**Relative effect, RR or MD (95% CI)**	**Participants, *n* (studies, *n*)**	**Certainty of the evidence (GRADE)**	**Comments**
**Primary outcome: number of participants achieving meaningful pain relief**					
Short term	2.7	RR 1.42 (1.08 to 1.89)	1700 (8)	+++– Moderate^a^ Due to limitations in study design	In the short term, opioids likely can provide meaningful pain relief to patients with OA or back pain with a number needed to treat of ~7, given 33.5% (*n* = 240/717) of control participants reported meaningful pain relief
Intermediate term	7.3	RR 1.04 (0.84 to 1.30)	900 (3)	++–– Low^b^ Due to imprecision and inconsistency	In the intermediate term, it is unclear whether opioids can provide meaningful pain relief to patients with OA or back pain
Long term	19.6	RR 0.91 (0.73 to 1.14)	3345 (9)	+++– Moderate^c^ Due to inconsistency in results	In the long term, it is unlikely that opioids provide meaningful pain relief to patients with OA or back pain and may even be counterproductive to pain management
**Secondary outcome: pain on a 100-point scale**					
Short term	—	MD –6.11 (–7.89 to –4.34)	5380 (18)	++++ High	In the short term, in patients with OA or back pain, opioids can reduce pain on a 100-point scale. On average, the magnitude of this reduction in pain is small
Intermediate term	—	MD –4.32 (–6.98 to –1.66)	2580 (7)	++++ High	In the intermediate term, in patients with OA or back pain, opioids can reduce pain on a 100-point scale. On average, the magnitude of this reduction in pain is small
Long term	—	MD –2.94 (–6.48 to 0.60)	4901 (12)	+++– Moderate^c^ Due to inconsistency in results	In the long term, in patients with OA or back pain, it is unclear whether opioids reduce pain on a 100-point pain scale

^a^Four of eight studies rated as ‘high’ risk of bias in ≥1 category; did not rate down for publication bias as average study size was relatively large (>200). ^b^High heterogeneity (*I* ^2 ^= 63%), with point estimates on either side of the line of no effect and 95% CIs barely overlapping; although optimal information size is met, the 95% CI includes no effect and the upper bound includes the preselected threshold for appreciable benefit (RR = 1.25). ^c^High heterogeneity (*I* ^2 ^= 81%), with point estimates on either side of the line of no effect and no overlap between some 95% CIs. GRADE = Grading of Recommendations Assessment, Development, and Evaluation. MD = mean difference. OA = osteoarthritis. RR = risk ratio.

**Figure 5.**
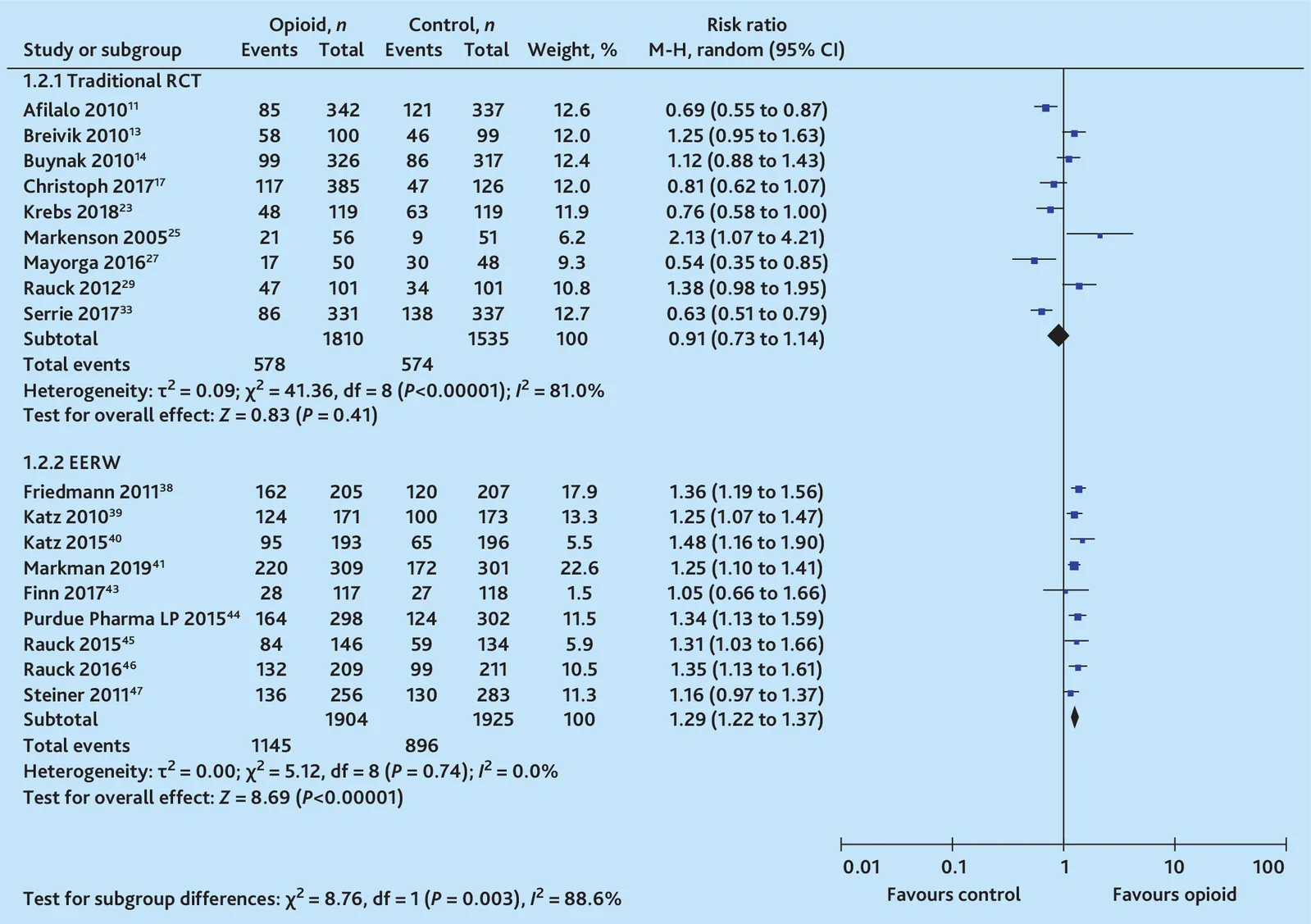
Sensitivity analysis examining the number of responders (the primary outcome) in long-term trials when an EERW trial design is employed compared with traditional RCTs. df = degrees of freedom. EERW = enriched enrolment randomised withdrawal. M-H = Mantel–Haenszel method. RCT = randomised controlled trial.

The sensitivity analysis, comparing the excluded EERW trials with the study’s included trials, was limited to nine trials with ≥12 weeks duration as only one EERW trial had a shorter duration. These EERW trials had an average opioid titration period of 5.6 weeks before randomisation to opioid withdrawal, and clinically significant pain relief was significantly more likely in the opioid group in EERW trials (risk ratio 1.29, 95% CI = 1.22 to 1.37), versus traditional RCTs (χ² = 8.76, df = 1, *P* = 0.003). A forest plot of this analysis is provided in Figure 5.

#### Secondary outcome: pain on a 100-point scale

Although there was a trend towards diminishing opioid benefit with time (Table 1 and Supplementary Figure S3), that trend was not statistically significant (χ² = 3.01, df = 2, *P* = 0.22), in part because differences in pain between groups were small at all timepoints. For all treatment durations, 95% CIs ruled out a mean difference of ≥8 on a 100-point scale.

## Discussion

### Summary

Compared with placebo or opioid-minimised treatment regimens, it is likely that clinically meaningful pain relief is more common in opioid recipients only in trials lasting ≤4 weeks, and trends in favour of control recipients for treatment durations of ≥12 weeks.

### Strengths and limitations

This study’s predefined choice of primary outcome, attainment of a minimum clinically important reduction in pain, limited the number of trials that could contribute to this analysis, as this outcome is not always reported. However, a responder analysis allowed the authors to pool trials that utilised different pain scales without sacrificing clinical meaning, and facilitated a bias-minimising intention-to-treat analysis given withdrawn or unaccounted for participants can be considered non-responders. The number of included trials was also limited by the study’s exclusion of trials with EERW designs. However, including such trials could have confounded the results given: a) the control groups were all initially exposed to opioids; b) only opioid responders were included in the trials; and c) withdrawing responders from opioids is unlikely to be adequately blinded.

Visual inspection of funnel plots for the primary and secondary outcomes clearly demonstrates the absence of smaller trials that are less favourable to opioids, indicating a strong publication bias. This bias, however, only strengthens the findings, as it runs contrary to the main finding that opioids may be without utility when used for longer durations. Although the analyses had high heterogeneity, this was expected given the wide range in specific opioids, dosages, durations, and controls, and heterogeneity was accounted for to some degree with a random-effects model.

Simultaneously, the findings are strengthened by a comprehensive and systematic search for eligible studies and a published protocol that predefined how the data would be analysed. They are also strengthened by the use of a responder analysis as a primary outcome (≥30% improvement), given this outcome aligns with patient-important change. It also permits all those who dropped out or were lost to follow-up (which are very common in opioid trials) to be treated as non-responders, thus reducing bias by accounting for unequal attrition or incomplete data, which can arise if one study arm provides inferior analgesia or more frequent or severe adverse effects.

### Comparison with existing literature

The current findings are consistent with studies supporting the existence of opioid tolerance and dependence, and of opioid-induced hyperalgesia.^1–3^ Evidence for the latter includes a systematic review of 26 non-randomised studies suggesting lower pain thresholds in participants who were chronic opioid users compared with control participants,^48^ and a systematic review of 27 RCTs demonstrating postoperative recovery periods with higher acute pain scores and higher total opioid consumption after a single intraoperative dose of a potent opioid.^49^ That a single dose of intraoperative opioid can influence pain perception during the postoperative recovery period helps support the decision to exclude EERW trial designs, in which the control participants are typically exposed to multiple weeks of opioid use during the trial run-in.

Contrary to the findings, two systematic reviews support opioids having superior pain relief over control treatment.^50,51^ However, both of these reviews included EERW trial designs. Given the seeming novelty of EERW designs, in the current study the authors went on to examine the trials included in two other systematic reviews of analgesics, one examining the use of acetaminophen to treat back pain and OA,^52^ and one examining the use of topical non-steroidal anti-inflammatory drugs (NSAIDs) to treat OA.^53^ Although the present opioid review found 10 of 37 (27%) trials otherwise eligible for the primary (responder) analysis to use the EERW design, none of the 49 acetaminophen or NSAID trials employed an EERW design, suggesting this design may be preferentially applied to the study of opioids.

### Implications for research and practice

The finding that opioids provide meaningful pain relief over a 4-week period, but have no benefit and perhaps even harm over longer durations, is clinically important. OA and chronic low back pain are long-term conditions. Patients motivated to start an opioid to manage those conditions will likely still have that same underlying painful condition a few weeks hence. For such individuals, limiting an intervention to a short course may not be practical and may lead to long-term use, resulting in risk of worse pain control, the potential for adverse drug effects, and possibly even substance use disorder. Key recommendations are therefore:

- healthcare providers should think twice before prescribing opioids for OA and chronic low back pain; and
- researchers should assess opioid efficacy at multiple timepoints, at least one of which should be ≥12 weeks*.*

The findings also raise concern regarding interpretation of EERW opioid trial results; in particular, concern that these trials are being included in meta-analyses seeking to estimate the analgesic effect of introducing opioids.^50,51^ Inclusion of EERW trials is particularly problematic given: a) EERW designs enrol only those who report meaningful analgesia during a 4–8-week opioid run-in period (thereby introducing opioid-favouring selection bias); and b) there is a high likelihood of unblinding when opioids are withdrawn in the control group post-randomisation. For RCTs ≥12 weeks in duration, the current study found markedly different (*P* = 0.003) effect estimates for the likelihood that opioids provide a ≥30% reduction in pain in trials with traditional designs (which suggest opioids likely provide no benefit and potentially even worsen pain control) and EERW designs (which suggest a 29% relative increase in meaningful analgesia). The EERW design is highly efficient (that is, such trials require fewer participants to demonstrate a difference), and likely encourages enrolment, given all participants can anticipate a minimum 4–8 weeks of opioid use regardless of allocation. However, the question being addressed in EERW trials is whether or not there is a maintenance effect in opioid responders. It is additionally problematic to equate differences in analgesia when opioids are withdrawn, to differences in analgesia when opioids are introduced, given tolerance and opioid-induced hyperalgesia after 4–8 weeks of opioid use could conceivably exacerbate pain in the control group on opioid discontinuation.

EERW trials are primarily used in the neuropsychiatric literature to assess durability of effect in conditions such as depression, bipolar affective disorder, schizophrenia, and epilepsy, where evidence of relapse post-discontinuation is being sought.^54,55^ Although it has been applied in the pain literature to evaluate the analgesic effect of pregabalin in neuropathic pain and fibromyalgia,^56,57^ the study found no examples of the EERW design being used to study NSAIDs or acetaminophen. Conceivably, the EERW design has utility in the early evaluation of a potential analgesic (that is, in ‘proof-of-concept’ trials), being statistically efficient in determining whether a subgroup exists for which benefit is possible. However, given this trial design overestimates benefit, and likely underestimates harm (as only those tolerating the drug will be randomised),^58^ the authors of the current study believe opioid trials employing an EERW design should not be combined in meta-analysis with other opioid trials when attempting to assess the value of introducing opioid analgesia.
